# Sonidegib Suppresses Production of Inflammatory Mediators and Cell Migration in BV2 Microglial Cells and Mice Treated with Lipopolysaccharide via JNK and NF-κB Inhibition

**DOI:** 10.3390/ijms231810590

**Published:** 2022-09-13

**Authors:** Ngoc Minh Nguyen, Men Thi Hoai Duong, Bich Phuong Bui, Phuong Linh Nguyen, Xiaozhen Chen, Jungsook Cho, Hee-Chul Ahn

**Affiliations:** College of Pharmacy, Dongguk University-Seoul, Goyang 10326, Korea

**Keywords:** sonidegib, hedgehog inhibitor, structure-based virtual screening, neuroinflammation, cell migration, c-Jun N-terminal kinase, nuclear factor-kappa B, drug repurposing, BV2 microglial cells

## Abstract

Our structure-based virtual screening of the FDA-approved drug library has revealed that sonidegib, a smoothened antagonist clinically used to treat basal cell carcinoma, is a potential c-Jun N-terminal kinase 3 (JNK3) inhibitor. This study investigated the binding of sonidegib to JNK3 via ^19^F NMR and its inhibitory effect on JNK phosphorylation in BV2 cells. Pharmacological properties of sonidegib to exert anti-inflammatory and anti-migratory effects were also characterized. We found that sonidegib bound to the ATP binding site of JNK3 and inhibited JNK phosphorylation in BV2 cells, confirming our virtual screening results. Sonidegib also inhibited the phosphorylation of MKK4 and c-Jun, the upstream and downstream signals of JNK, respectively. It reduced the lipopolysaccharide (LPS)-induced production of pro-inflammatory factors, including interleukin-1β (IL-1β), IL-6, tumor necrosis factor-α (TNF-α), and nitric oxide (NO), and the expression of inducible NO synthase and cyclooxygenase-2. The LPS-induced cell migration was suppressed by sonidegib. Sonidegib inhibited the LPS-induced IκBα phosphorylation, thereby blocking NF-κB nuclear translocation. Consistent with these findings, orally administered sonidegib attenuated IL-6 and TNF-α levels in the brains of LPS-treated mice. Collectively, our results indicate that sonidegib suppresses inflammation and cell migration in LPS-treated BV2 cells and mice by inhibiting JNK and NF-κB signaling. Therefore, sonidegib may be implicated for drug repurposing to alleviate neuroinflammation associated with microglial activation.

## 1. Introduction

Neuroinflammation is a common feature of many neurodegenerative diseases, including Alzheimer’s disease (AD) and Parkinson’s disease (PD). Thus, controlling neuroinflammatory processes has emerged as a promising therapeutic option. In such strategies, glial cells rather than neurons act as cellular targets [1]. Microglia function as the first line of immune defense within the central nervous system (CNS) and major players in neuroinflammation [2]. Therefore, biological knowledge of this cell type is pivotal in understanding the pathology of CNS diseases. An increasing amount of evidence has shown that microglial activation is an early and ongoing event in several neurodegenerative diseases. Overactivated microglia is a chronic source of multiple neurotoxic factors, including interleukin-1β (IL-1β), tumor necrosis factor-α (TNF-α), nitric oxide (NO), and reactive oxygen species (ROS), which contribute to neuronal damage [3,4,5,6]. Hence, suppression of microglial activation may be a useful therapeutic strategy to alleviate neuroinflammation.

c-Jun N-terminal kinases (JNKs), which belong to the superfamily of mitogen-activated protein kinases (MAPKs), are stress-response elements. The JNK pathway is a promising therapeutic target controlling a wide range of cellular mechanisms under physiological and pathological conditions [7]. This pathway is also considered a critical intermediate signaling in the immune system [8]. Stress stimuli, such as cytokines and irradiation, activate JNKs through dual phosphorylation in threonine (Thr) and tyrosine (Tyr) residues by two MAPK kinases, MKK4 and MKK7, with a JNK-interacting scaffold protein [7]. Subsequently, JNKs phosphorylate and activate the transcription factor c-Jun and potentiate its transcriptional activity [9,10]. Among three different JNK isoforms, JNK3 is almost exclusively expressed in the CNS [11,12]. It plays critical roles in the pathogenesis of many neurological disorders. Selective expression of JNK3 in the CNS may render neurons particularly vulnerable to environmental stress. *JNK3*-knockout mice exhibited a remarkable resistance to seizures and apoptosis induced by the excitotoxic glutamate-receptor agonist kainic acid [13]. Moreover, JNK3 is required for 1-methyl-4-phenyl-1,2,4,6-tetrahydropyridine (MPTP)-induced c-Jun activation and dopaminergic cell death. Elimination of JNK3 not only protects dopaminergic neurons against MPTP-induced neurodegeneration, but also improves the motor function in this animal model of PD [14]. The inhibition of the JNK3/PI3K/AKT pathway by *Momordica charantia* polysaccharide protects against behavioral defects and decreases the expression of pro-inflammatory cytokines induced by chronic social defeat stress in an animal model [15]. Therefore, JNK3 is a specific key modulator in degenerative conditions in the brain.

Drug repositioning is defined as a process through which new therapeutic uses, in addition to original indications, of approved drugs are discovered [16]. Through this approach, drugs can be more rapidly developed with reduced cost and risk [17]. To identify potential JNK3 inhibitors from the library of drugs approved by the U.S. Food and Drug Administration (FDA), we performed structure-based virtual screening by using the three-dimensional (3D) structure of JNK3. Among the drugs screened, sonidegib, a selective smoothened (SMO) antagonist approved for the treatment of advanced basal cell carcinoma [18], is one of the potential JNK3 inhibitors. With its favorable ability to penetrate the blood–brain barrier (BBB), sonidegib is currently under active clinical trials involving children and adults with relapsed medulloblastoma [19]. Recent studies have demonstrated that Sonic hedgehog signaling is critical for the recruitment of inflammatory cells during infection in the stomach and immune quiescence in the brain; these results indicate the potential role of this pathway in neuroinflammation [20,21]. Moreover, an SMO mutant has been identified as a genetic modifier of amyloid beta (Aβ) toxicity, and downregulation of SMO alleviates Aβ-activated innate inflammatory responses in flies [22]. The pharmacological inhibition of SMO by sonidegib rescues Aβ-induced memory loss in a Morris water maze. In a mouse model of AD, the number of astrocytes secreting pro-inflammatory factors, such as IL-1β and TNF-α, and the expression of microglia marker Iba-1 are reduced [22]. Furthermore, sonidegib significantly reduces the activation of macrophages and the expression of TNF-α, IL-1β, IL-6, and monocyte chemoattractant protein 1 in high-fat diet-induced non-alcoholic fatty liver diseases [23]. These findings strongly support the possibility of repositioning of sonidegib to various central as well as peripheral disorders associated with inflammation.

In addition to the JNK pathway, nuclear factor-kappa B (NF-κB) plays a central role in the regulation of the inflammatory response [24,25]. The activation of NF-κB is required for the transcriptional induction of many pro-inflammatory mediators involved in innate immunity [26]. These mediators include IL-6, inducible nitric oxide synthase (iNOS), cyclooxygenase-2 (COX-2), and matrix metalloproteinase-9. A previous study has found that NF-κB is overactivated in microglial cells after ischemic stroke [27]. Therefore, inhibition of neuroinflammation via the suppression of NF-κB activity in microglia has emerged as a breakthrough target for the treatment of various brain disorders, including ischemic stroke [26].

In this study, we verified the binding of sonidegib to JNK3 via ^19^F NMR and examined its inhibitory effect on JNK phosphorylation in BV2 microglial cells exposed to lipopolysaccharide (LPS). We also investigated the anti-inflammatory and anti-migratory effects of sonidegib through the inhibition of JNK and NF-κB activity. We further evaluated the anti-inflammatory effect of orally administered sonidegib in LPS-treated ICR mice. Our findings showed that sonidegib may be a promising agent to alleviate neuroinflammation mediated by JNK and NF-κB signaling.

## 2. Results

### 2.1. Sonidegib Binds to JNK3 in an ATP-Competitive Manner

**Figure 1:**
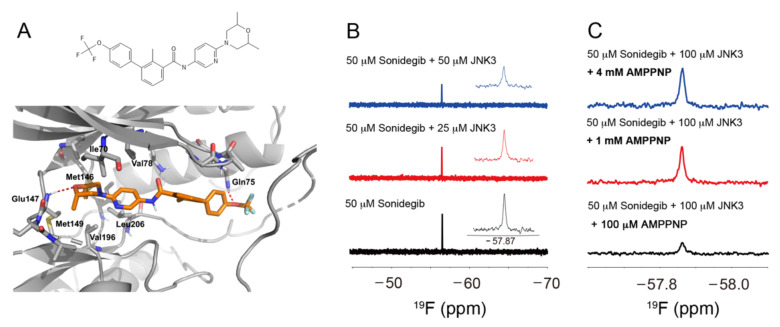
Binding of sonidegib to JNK3. (**A**) Chemical structure of sonidegib and proposed binding of sonidegib to JNK3 via docking simulation. JNK3 and sonidegib are displayed as a gray cartoon and orange sticks, respectively. Hydrogen bonds are expressed as red broken lines, and the residues in close contact with sonidegib are highlighted as gray sticks. (**B**) ^19^F NMR spectra of 50 μM sonidegib in the absence (black) or presence of 25 and 50 μM JNK3 (red and blue, respectively). (**C**) ^19^F NMR spectra of 50 μM sonidegib and 100 μM JNK3 in the presence of AMPPNP, a non-cleavable ATP analog, at the final concentrations of 0.1, 1, and 4 mM (black, red, and blue, respectively).

To identify potential JNK3 inhibitors from the FDA-approved drug library of 2580 compounds, we performed structure-based virtual screening by using the 3D structure of JNK3 retrieved from Protein Data Bank (PDB) (http://www.rcsb.org). Chemical structure of sonidegib and the lowest energy structure from the docking simulation of sonidegib to JNK3 is shown in Figure 1A. The simulation score was −10.4 kcal/mol. In the simulated structure, sonidegib binds to the ATP binding site of JNK3, and a hydrogen bond forms between the oxygen of sonidegib and the backbone –NH of Glu147 in JNK3. Glu147 is an important residue in ATP binding because it forms a hydrogen bond with the adenine ring of ATP [28]. Other JNK3 residues that are likely essential for its binding to sonidegib (Figure 1A) include several hydrophobic residues, such as Ile70, Val78, Met146, Met149, Val196, and Leu206. One more hydrogen bond was found on the other end of sonidegib between –OCF_3_ and –NH of Gln75.

To verify the binding of sonidegib to JNK3, we conducted ^19^F NMR spectroscopy. The chemical shift of –CF_3_ group in the sonidegib molecule was −57.87 ppm (Figure 1B). After we added 25 or 50 μM JNK3 to sonidegib, the ^19^F signal in –CF_3_ decreased in a concentration-dependent manner primarily because of the enhancement of ^19^F relaxation by the binding of the compound to the large protein JNK3 (Figure 1B). The ^19^F signal intensities of sonidegib were restored when we added AMPPNP, a non-cleavable ATP analog, to the mixture of sonidegib and JNK3 (Figure 1C). In the presence of 1 mM AMPPNP, which was 20-fold higher than the sonidegib concentration, the ^19^F signal intensity was comparable with that of sonidegib alone. These results implied that AMPPNP competes with sonidegib on the same site of JNK3 and repels the pre-bound sonidegib from the ATP binding site of JNK3. Therefore, sonidegib binds to the ATP binding site of JNK3 and functions as an ATP-competitive inhibitor.

### 2.2. Sonidegib Inhibits the LPS-Induced Phosphorylation of JNK, c-Jun, and MKK4 in BV2 Cells

**Figure 2:**
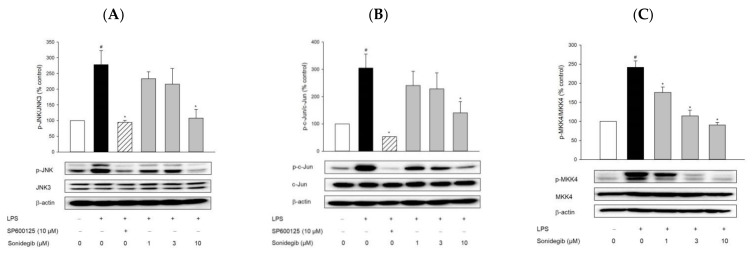
Effects of sonidegib on the LPS-induced phosphorylation of JNK, c-Jun, and MKK4 in BV2 cells. Cells were co-treated with LPS (1 μg/mL) and SP600125 (10 μM) or a series of sonidegib concentrations for 24 h. Western blot analysis was performed as described in the Materials and Methods to assess the phosphorylation levels with anti-phospho-JNK (p-JNK) and JNK3 antibodies (**A**), anti-phospho-c-Jun (p-c-Jun) and c-Jun antibodies (**B**), and anti-phospho-MKK4 (p-MKK4) and MKK4 antibodies (**C**). β-Actin was chosen as a loading control. Representative blots are provided. Data are expressed as the mean ± SEM from at least three independent experiments. # *p* < 0.05 vs. vehicle-treated control cells and * *p* < 0.05 vs. LPS-treated cells. The experimental conditions for the bars in the graph are indicated under the corresponding blots in each figure.

To verify the structure-based virtual screening results by using BV2 microglial cells as a cellular model, we first examined the effect of sonidegib on LPS-induced JNK phosphorylation. We treated the cells with sonidegib at the indicated concentrations in the presence of LPS for 24 h and performed Western blot analysis with the specific antibodies recognizing phospho-JNK or JNK3. Figure 2A shows that 10 μM sonidegib significantly inhibited LPS-induced JNK phosphorylation, verifying our previous virtual screening results. The inhibition of JNK phosphorylation by sonidegib was comparable with that by SP60025, a well-known JNK inhibitor. We then examined the effects of sonidegib on the LPS-induced phosphorylation of c-Jun and MKK4, which are downstream and upstream molecules of JNK, respectively. We observed that sonidegib inhibited the LPS-induced phosphorylation of c-Jun and MKK4 in BV2 cells (Figure 2B,C).

### 2.3. Sonidegib Suppresses LPS-Stimulated Migration in BV2 Cells

**Figure 3:**
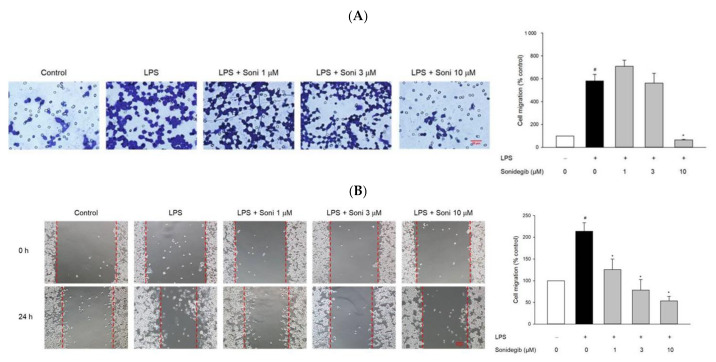
Effect of sonidegib on LPS-stimulated BV2 cell migration. Cells were co-treated with LPS (1 μg/mL) and a series of sonidegib concentrations for 24 h, and Transwell migration (**A**) and wound healing (**B**) assays were performed as described in the Materials and Methods. The representative images are shown. Scale bar, 20 μm. Data are expressed as the mean ± SEM from at least three independent experiments. # *p* < 0.05 vs. vehicle-treated control cells and * *p* < 0.05 vs. LPS-treated cells. Soni, sonidegib. 1000.

Directed cell migration after microglial activation, resulting in cell accumulation at injury sites, is crucial to the immune responses of microglial cells and may contribute to the pathogenesis of brain diseases [29]. Therefore, we next examined the effect of sonidegib on cell migration stimulated with LPS in BV2 cells via Transwell migration and wound healing assays. Upon LPS treatment, marked increases in the number of migrated cells were observed in both assays, as compared with the control groups (Figure 3). The LPS-stimulated BV2 cell migration was dramatically suppressed by sonidegib at the concentration of 10 μM. In the wound healing assay, LPS-stimulated cell migration was significantly inhibited by sonidegib at a concentration of as low as 1 μM (Figure 3B).

### 2.4. Sonidegib Inhibits the LPS-Induced Production of Pro-Inflammatory Factors in BV2 Cells

**Figure 4:**
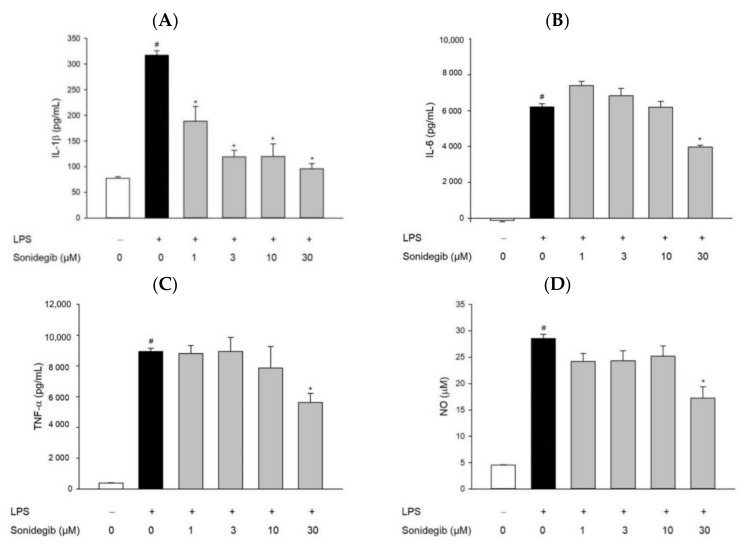
Effects of sonidegib on the LPS-induced production of pro-inflammatory factors in BV2 cells. Cells were co-treated with LPS (1 μg/mL) and a series of sonidegib concentrations for 24 h. After the treatment, culture media were collected, and the levels of pro-inflammatory mediators, including IL-1β (**A**), IL-6 (**B**), TNF-α (**C**), and NO (**D**), were determined using ELISA kits and Griess assay as described in the Materials and Methods. Data are obtained from at least three independent experiments and expressed as the mean ± SEM. # *p* < 0.05 vs. vehicle-treated control cells and * *p* < 0.05 vs. LPS-treated cells.

To assess the anti-inflammatory effect of sonidegib, we subsequently tested its impact on the LPS-induced production of pro-inflammatory mediators in BV2 cells. We co-treated the cells with LPS and a series of concentrations of sonidegib for 24 h and measured the levels of inflammatory mediators in the culture media by using ELISA kits and Griess assay. Our data showed that LPS treatment markedly induced the production of IL-1β (Figure 4A), IL-6 (Figure 4B), TNF-α (Figure 4C), and NO (Figure 4D) in BV2 cells. The simultaneous treatment with LPS and sonidegib significantly reduced the production of these factors, demonstrating anti-inflammatory action. While the LPS-induced production of IL-1β was prominently inhibited by sonidegib at all tested concentrations (Figure 4A), the production of IL-6, TNF-α, and NO was only significantly reduced by sonidegib at 30 μM (Figure 4B–D).

### 2.5. Sonidegib Inhibits the LPS-Induced Expression of iNOS and COX-2 in BV2 Cells

**Figure 5:**
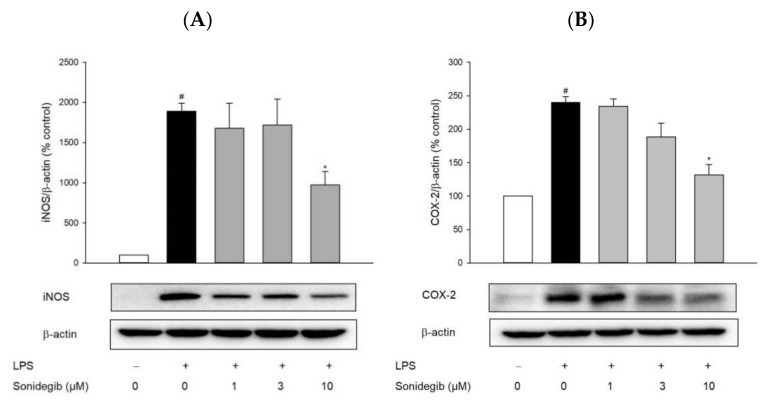
Effects of sonidegib on the LPS-stimulated expression of iNOS and COX-2 in BV2 cells. Cells were co-treated with LPS (1 μg/mL) and a series of sonidegib concentrations for 24 h. The expression levels of iNOS (**A**) and COX-2 (**B**) were examined by Western blot analysis, as described in the Materials and Methods. β-Actin was chosen as a loading control. Representative blots are shown. Data are expressed as the mean ± SEM from at least three independent experiments. # *p* < 0.05 vs. vehicle-treated control cells and * *p* < 0.05 vs. LPS-treated cells. The experimental conditions for the bars in the graph are indicated under the corresponding blots in each figure.

To clarify the underlying mechanisms by which sonidegib exerts the anti-inflammatory action, we evaluated the effects of sonidegib on the levels of pro-inflammatory enzymes iNOS and COX-2 via Western blot analysis. We found that the iNOS and COX-2 expression levels were markedly increased by LPS treatment. These increased expression levels were reduced by 10 µM sonidegib (Figure 5).

### 2.6. Sonidegib Reduces the LPS-Induced Nuclear Translocation of NF-κB in BV2 Cells

**Figure 6:**
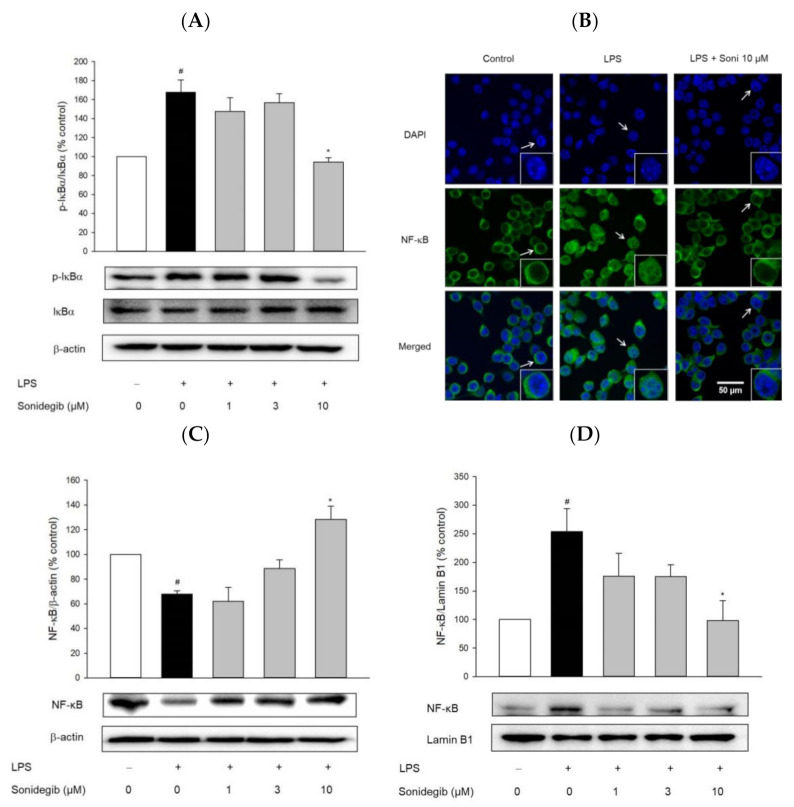
Effects of sonidegib on the LPS-induced phosphorylation of IκBα and nuclear translocation of NF-κB in BV2 cells. Cells were treated with various concentrations of sonidegib in the presence of LPS (1 μg/mL) for 24 h. (**A**) The expression levels of phospho- and non-phospho-IκBα were evaluated via Western blotting, as described in the Materials and Methods, by using antibodies specifically recognizing the respective protein. β-Actin was chosen as a loading control. Data are expressed as the mean ± SEM from at least three independent experiments. (**B**) Cells were co-treated with LPS (1 µg/mL) and sonidegib (10 µM) for 24 h, and immunocytochemical analysis was conducted to visualize the localization of NF-κB by using DAPI (blue color) and anti-NF-κB p65 antibody (green color), as described in the Materials and Methods. Representative images are shown. The arrow in each image indicates the magnified cell shown in the inset. Scale bar, 50 µm. The levels of cytosolic (**C**) and nuclear (**D**) NF-κB were evaluated via Western blot by using anti-NF-κB p65 antibody in cytosolic and nuclear fractions. β-Actin and lamin B1 were chosen as loading controls for the cytosolic and nuclear fractions, respectively. Representative blots are shown. Data are expressed as the mean ± SEM from at least three independent experiments. # *p* < 0.05 vs. vehicle-treated control cells and * *p* < 0.05 vs. LPS-treated cells. The experimental conditions for the bars in the graph are indicated under the corresponding blots in each figure.

In activated glial cells, the transcription factor NF-κB initiates various inflammatory processes that contribute to the pathology of neurodegeneration. Phosphorylation of inhibitory kappa B (IκB) and its subsequent degradation are important events in NF-κB activation in response to different stimuli [30]. Therefore, the effects of sonidegib on the LPS-induced IκBα phosphorylation and NF-κB translocation were examined in BV2 cells to determine whether the anti-inflammatory effect of sonidegib was associated with NF-κB activation. The cells exposed to LPS increased IκBα phosphorylation, which was significantly reversed by 10 μM sonidegib (Figure 6A). Consistent with this finding, immunocytochemical and Western blot analyses demonstrated that sonidegib inhibited the LPS-induced nuclear translocation of NF-κB (Figure 6B–D). Therefore, NF-κB possibly contributed to the anti-inflammatory effect of sonidegib in LPS-treated activated microglial cells.

### 2.7. Sonidegib Reduces the Production of IL-6 and TNF-α in the Brains of LPS-Treated Mice

**Figure 7:**
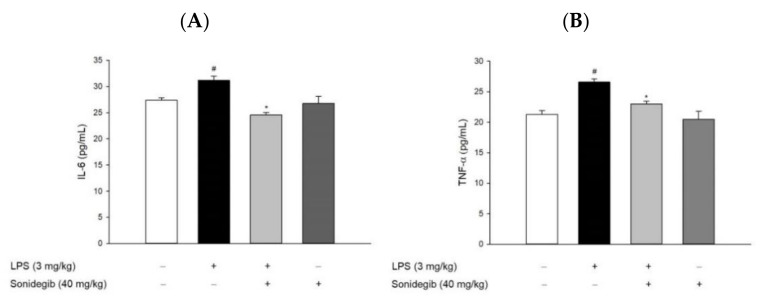
Effects of sonidegib on IL-6 and TNF-α levels in the brain tissues of LPS-treated mice. ICR mice (8 mice per each group) were orally administered with sonidegib or vehicle at a dosage of 40 mg/kg once daily for 7 days and challenged with intraperitoneally injected LPS (3 mg/kg) on the 7th day. Brains were dissected after 24 h of LPS injection and homogenized in lysis buffer. Then, the levels of IL-6 (**A**) and TNF-α (**B**) were determined in the brain homogenates by using ELISA kits, as described in the Materials and methods. Data are expressed as the mean ± SEM in each group. # *p* < 0.05 vs. vehicle-treated control group and * *p* < 0.05 vs. LPS-treated group without sonidegib administration.

To assess anti-inflammatory activity of sonidegib in vivo, ICR mice were orally administered with sonidegib or vehicle at a dosage of 40 mg/kg once daily for 7 days. On the 7th day, neuroinflammation was induced by intraperitoneal injection of LPS (3 mg/kg) 1 h after the final administration of sonidegib [31]. Then, the levels of IL-6 and TNF-α were determined in the homogenates of brain tissues obtained from the sacrificed mice. As presented in Figure 7, the IL-6 and TNF-α levels slightly, but significantly, increased in the brains of LPS-injected mice. Consistent with our findings in BV2 cells (Figure 4B,C), the oral administration of sonidegib reversed the levels of these pro-inflammatory cytokines up to the levels comparable with those in the vehicle-treated control group (Figure 7A,B), demonstrating manifest anti-inflammatory effect in mice. The IL-6 and TNF-α levels in the mice treated with sonidegib alone were not different from those in the control mice.

## 3. Discussion

Considering the potential advantages of high efficiency, low cost, and less risk, drug repositioning has recently become a popular strategy to assign new indications to approved drugs [32]. As the second SMO antagonist, sonidegib was approved in 2015 for the treatment of advanced basal cell carcinoma. Subsequent studies including clinical trials are in progress to expand its application to other types of cancer [18,19,33]. Apart from its role in cancer therapies, sonidegib was shown to exhibit protective effects in several animal models of CNS disorders, such as AD and ischemic stroke, by inhibiting Aβ-induced memory deficits, neuroinflammation, and glutamate toxicity [22,34]. In our preliminary study involving the structure-based virtual screening of the FDA-approved drug library with 2580 compounds, we identified several drugs, including azelastine, efonidipine, and sonidegib, as potential JNK3 inhibitors. In accordance with these findings, azelastine (an anti-histamine drug) and efonidipine (a calcium channel blocker) were demonstrated to exert anti-inflammatory and anti-migratory activities in BV2 cells through inhibition of JNK [35], providing potential evidence for repurposing of these drugs. In the current study, we report that sonidegib binds to the ATP binding site of JNK3, thereby inhibiting the LPS-induced JNK phosphorylation in BV2 cells. These findings verified our virtual screening results. Moreover, we report suppression of inflammatory mediators and cell migration by sonidegib in LPS-treated BV2 cells and mice by inhibiting JNK and NF-κB. These findings also support the potential repurposing of this agent to alleviate neuroinflammation associated with many brain disorders. However, the therapeutic potential of sonidegib in various animal models or even in patients should be further evaluated.

JNKs have essential pro-inflammatory functions in microglia; thus, JNK inhibition may elicit anti-inflammatory effects on microglial activation [5]. In particular, JNK3, the brain-specific subtype of JNK, is implicated in several neurodegenerative diseases, including AD, PD, and stroke [36]. Indeed, inhibition of JNK3 phosphorylation effectively attenuates synaptic dysfunction, inflammatory responses, and multiple neuropathologies associated with AD [37]. Conversely, activation of JNK3 is involved in the rotenone-induced apoptosis of dopaminergic neurons, a typical experimental model mimicking the pathogenesis of PD [38]. By blocking JNK3 signaling, the main bioactive ingredient of *M. charantia* suppresses free radical elevation and subsequently inhibits delayed neuronal cell death, thereby exerting a neuroprotective function after intra-cerebral hemorrhagic injury [39]. These studies have suggested that JNK3 may be a promising target to mitigate neuroinflammation associated with neurodegenerative brain disorders. Our virtual screening and docking simulation demonstrated that sonidegib bound to the ATP binding site of JNK3 and inhibited the JNK3 activity. It showed strong affinity to the binding site with a simulation score of −10.4 kcal/mol. The binding of sonidegib to JNK3 was also authenticated via ^19^F NMR spectroscopy. The experiments with AMPPNP further confirmed the role of sonidegib as an ATP-competitive inhibitor of JNK3 (Figure 1).

Among numerous downstream targets of JNK, c-Jun is the major substrate of JNK; thus, it serves as a JNK-specific biomarker [40]. As a transcription factor, c-Jun also represents a pivotal role in regulating neuronal and glial responses to injury [41]. In the current study, our virtual screening results were confirmed by evaluating the effect of sonidegib on JNK phosphorylation in LPS-treated BV2 microglial cells. Indeed, sonidegib inhibited the LPS-induced phosphorylation of JNK in BV2 cells (Figure 2A). The inhibition by sonidegib was nearly comparable with that by the positive reference SP600125, a well-known JNK inhibitor. In addition, the LPS-induced phosphorylation of c-Jun was also inhibited by sonidegib (Figure 2B). Two protein kinases that activate JNK have been identified as MKK4 (or JNKK1, SEK1) and MKK7 (or JNKK2). Although both kinases activate JNK, MKK4 phosphorylates JNK preferentially on Tyr, whereas MKK7 prefers Thr [42]. In addition, MKK4 is activated primarily by environmental stress or mitogenic stimuli, and MKK7 is mainly triggered by cytokines [43]. Our study found that MKK4 phosphorylation in BV2 cells was strongly induced in response to LPS treatment, while this effect was inhibited by sonidegib (Figure 2C). Meanwhile, LPS treatment failed to cause MKK7 phosphorylation in BV2 cells. Therefore, sonidegib could inhibit the LPS-induced MKK4/JNK/c-Jun signaling pathway.

Neuroinflammation refers to a complex inflammatory response caused by various pathological insults, including infection, trauma, ischemia, and toxins, in the CNS [44]. Among innate immune cells, microglia are the primary players in neuroinflammation. Under pathological conditions, activated microglia, along with astrocytes and macrophages migrating through the damaged BBB, accumulate at the injured sites, ultimately contributing to the pathogenesis of brain diseases [29]. In our study, we found that LPS markedly stimulated the migration of microglial cells. Meanwhile, sonidegib inhibited BV2 cell migration induced by LPS treatment, which were confirmed from both Transwell migration assay and wound healing assay (Figure 3). The activated microglial cells lose their homeostatic functions and produce increased amounts of pro-inflammatory cytokines and chemokines, leading to synaptic dysfunction and neuronal death [45,46]. For example, IL-1β induces synaptic loss by increasing prostaglandin E2 production, thereby releasing glutamate and activating *N*-methyl-D-aspartate receptor [47]. TNF causes neuronal death by activating TNF receptor 1 and recruiting caspase 8 when the NF-κB pathway is inhibited [48]. IL-6 mediates neuroinflammation and contributes to motor coordination deficits after mild traumatic brain injury [49]. During microglial activation, activated NADPH oxidase, iNOS, and COX-2 result in the synthesis of high levels of ROS, NO, and PGE2; consequently, oxidative stress is enhanced to exert toxic effects on dopaminergic neurons, further amplifying the pro-inflammatory microenvironment [50]. Because of the involvement of activated microglia in neuroinflammation, the suppression of microglial activation is considered a therapeutic option for neurodegenerative diseases. In our study, sonidegib manifested an anti-inflammatory effect by inhibiting the LPS-induced production of pro-inflammatory cytokines, including IL-1β, IL-6, and TNF-α (Figure 4A–C), and NO (Figure 4D). Consistent with these findings, sonidegib also significantly inhibited the LPS-induced protein expression of iNOS and COX-2 (Figure 5), two important inducible pro-inflammatory enzymes. Therefore, sonidegib could suppress microglial activation, implying its potential therapeutic effects against various neuroinflammatory responses.

The activation of the NF-κB family of transcription factors is a key step in the regulation of inflammatory and immune responses [51]. Through an NF-κB-mediated mechanism, microglia produce large amounts of pro-inflammatory cytokines and ROS, which may indirectly promote neuronal death [52,53]. Accordingly, NF-κB has become a molecular target of therapeutic anti-inflammatory agents [54]. In resting cells, NF-κB dimers are retained in the cytosol by the association with the inhibitory proteins of the IκB family. The crucial step in NF-κB activation is the phosphorylation of IκB proteins by the IκB kinase complex. The phosphorylation of inhibitory IκB proteins initiates their ubiquitination and undergoes subsequent proteosomal degradation, while the released NF-κB translocates to the nucleus and induces the expression of its target genes [51]. In the present study, LPS treatment induced IκBα phosphorylation and subsequently induced NF-κB translocation from the cytosol to the nucleus. Western blot and immunocytochemical analyses indicated that the LPS-induced nuclear translocation of NF-κB was markedly abolished by sonidegib in BV2 cells (Figure 6). These findings demonstrate that the inactivation of NF-κB by sonidegib also contributes, at least in part, to its anti-inflammatory properties in LPS-treated BV2 cells.

Since JNKs, with a special emphasis on JNK3, are often targeted for the treatment of CNS disorders, potential drugs should be tested for their ability to penetrate the brain [40]. Sonidegib can favorably penetrate the BBB and is under active clinical trials in brain tumors [19,55]. In the present study, we evaluated the anti-inflammatory effects of sonidegib in vivo by using a mouse model of acute brain inflammation induced by LPS injection. Intraperitoneal injection of LPS to mice is one of the most important and widely used animal models to induce neuroinflammation [31,56,57]. Our data showed that sonidegib significantly inhibited IL-6 and TNF-α release in the brains of LPS-treated mice (Figure 7). These results were consistent with our in vitro observations in BV2 cells.

**Figure 8:**
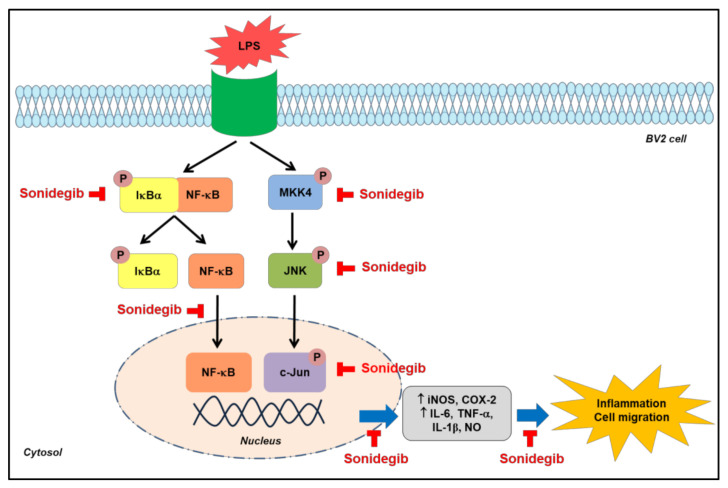
A schematic diagram illustrating the anti-inflammatory and anti-migratory effects of sonidegib and the molecules mediating its effects in LPS-treated BV2 cells and mice. The signaling molecules inhibited by sonidegib are indicated with red blocked arrows. LPS, lipopolysaccharide; IκBα, inhibitory kappa Bα; NF-κB, nuclear factor-kappa B; MKK4, mitogen-activated protein kinase kinase 4; JNK, c-Jun N-terminal kinase; iNOS, inducible nitric oxide synthase; COX-2, cyclooxygenase-2; IL-6, interleukin-6; TNF-α, tumor necrosis factor-α; IL-1β, interleukin-1β; NO, nitric oxide.

Abnormal microglial activation has been associated with the production of pro-inflammatory enzymes, cytokines, and chemokines, which are closely associated with many neurodegenerative disorders. Thus, understanding the underlying mechanisms to control microglial activation and pro-inflammatory mediators is crucial in the development of new treatment strategies for neurodegenerative disorders. Our results demonstrated the anti-inflammatory role of sonidegib through its inhibition of pro-inflammatory enzymes (iNOS and COX-2), production of cytokines (TNF-α, IL-1β, and IL-6), and NO. It also showed suppression of LPS-induced BV2 cell migration. Collectively, the inhibition of MKK4/JNK/c-Jun signaling as well as NF-κB nuclear translocation by sonidegib are identified to mediate its anti-inflammatory and anti-migratory effects, as illustrated in Figure 8. Although our data presented in the current study fully supported our conclusion, additional experiments with small interfering RNA (siRNA) to knockdown JNK or NF-κB, the major signaling molecules shown to mediate the effects of sonidegib, would be useful to further strengthen our observation.

## 4. Materials and Methods

### 4.1. Chemicals and Reagents

The following substances were used in this study: Dulbecco’s modified Eagle medium (DMEM), fetal bovine serum (FBS), and antibiotic–antimycotic agent (Corning Life Science, Corning, NY, USA); sonidegib (Selleckchem, Houston, TX, USA); SP600125 (Calbiochem, Darmstadt, Germany); LPS from *Escherichia coli* (serotype O111:B4), dimethyl sulfoxide (DMSO), and anti-β-actin and anti-lamin B1 antibodies (Sigma-Aldrich, St. Louis, MO, USA); goat anti-mouse IgG secondary antibody conjugated with Alexa Fluor 488 and 4′,6-diamidino-2-henylindole dihydrochloride (DAPI; Thermo Scientific, Rockford, IL, USA); fluorescent mounting medium (Dako, Carpinteria, CA, USA); MKK4 antibody (Santa Cruz Biotechnology, Dallas, TX, USA); anti-phospho-IκBα antibody (Abcam, Cambridge, MA, USA); and primary antibodies against phospho-MKK4, phospho-JNK, JNK3, phospho-c-Jun, c-Jun, iNOS, COX-2, NF-κB p65, and IκBα and secondary anti-rabbit and anti-mouse antibodies (Cell Signaling Technology, Danvers, MA, USA).

### 4.2. Docking Simulation and NMR Spectroscopy

Virtual screening and molecular docking simulation were conducted as described in our previous work [35]. The catalytic domain of JNK3 was prepared as previously described [58]. Sonidegib was prepared in a solution containing 50 mM sodium phosphate at pH 7.0, 0.3% Tween 20, 4% DMSO, and 10% D_2_O and used at the final concentration of 50 μM in NMR experiments as indicated. JNK3 concentrations were from 25 to 100 μM. For competition experiments, AMPPNP was titrated to the mixture of sonidegib and JNK3 until the final concentrations of 100 μM, 1 mM, and 4 mM were obtained. The ^19^F NMR data were acquired at 25 °C by using an Agilent DD2 600 MHz NMR spectrometer with oneNMR™ probe tuned to ^19^F frequency of 564.462 MHz. The sweep widths were 28,409.1 Hz, and the time-domain points were 32K for all experiments. Data were processed and analyzed with Mnova (Mes-trelab Research, Santiago de Compostela, Spain).

### 4.3. Cell Culture and Treatment

BV2 microglial cells were maintained in DMEM supplemented with 10% heat-inactivated FBS and 1% antibiotic-antimycotic agent at 37 °C in a humidified incubator under 5% CO_2_ [5]. LPS was dissolved in the distilled water to a stock solution of 1 mg/mL. Sonidegib and SP600125 were dissolved in DMSO to 10 mM stock solution and stored at –20 °C. Cells were treated with LPS (1 µg/mL) with or without various concentrations of sonidegib or SP600125 (10 µM) for 24 h.

### 4.4. Western Blot Analysis

BV2 cells (1 × 10^6^ cells/dish) were seeded on 35 mm culture dishes and treated with different sonidegib concentrations in the presence of LPS (1 µg/mL) for 24 h. After the desired treatment, the cells were washed with cold phosphate-buffered saline (PBS) and lysed in lysis buffer, as described previously [59]. To extract cytosolic and nuclear proteins, BV2 cells (2.5 × 10^6^ cells/dish) were plated on 60 mm culture dishes and co-treated with LPS and sonidegib at the indicated concentrations. After the treatment, the cells were washed with cold PBS, and cytosolic and nuclear proteins were obtained according to the NE-PER ^®^ instructions (Thermo Fisher, Rockford, IL, USA). The protein concentration in the lysates was measured using a Bio-Rad DC protein assay kit (Bio-Rad, Hercules, CA, USA). An equal amount of protein was separated through sodium dodecyl sulfate-polyacrylamide gel electrophoresis (SDS-PAGE) and subsequently transferred to polyvinylidene fluoride (PVDF) membranes (Merck Millipore Ltd., Billerica, MA, USA). The membranes were blocked with 5% skim milk solution at room temperature for 1 h and probed with specific primary antibodies at 4 °C overnight. Then, appropriate horseradish peroxidase-conjugated secondary antibodies were added and incubated with the membranes at room temperature for 2 h. The signals were measured with an enhanced chemiluminescent (ECL) reagent and ChemiDoc XRS imaging system (Bio-Rad, Hercules, CA, USA).

### 4.5. Cell Migration Assays

#### 4.5.1. Transwell Migration Assay

BV2 cells (1 *×* 10^5^ cells/well) were placed on the upper layer of a Transwell insert with a permeable membrane (6.5 mm in diameter and 8.0 µm in pore size) from Costar (Corning Inc., Kennebunk, ME, USA). After 24 h of incubation at 37 °C, treatment solution with LPS (1 µg/mL) and sonidegib at the indicated concentrations was added to the bottom of the lower chamber. After 24 h of treatment, the Transwell inserts were placed in 4% paraformaldehyde for 20 min to allow cell fixation, permeabilized with methanol for 10 min, and stained with 0.5% crystal violet for 10 min. The cells in the upper chamber of the insert were then gently removed using a cotton swab, and the insert was visualized under a phase-contrast microscope (Nikon Instruments Inc., Melville, NY, USA). The migrated cells were counted in at least five different fields, and the average was calculated. The degree of cell migration was described as the percentage of vehicle-treated control cells [5].

#### 4.5.2. Wound Healing Assay

BV2 cells (5 *×* 10^5^ cells/well) were seeded into 24-well plates and incubated for 24 h. The cells were scratched with a sterile scratcher (SPL, Korea) and treated with 1, 3, and 10 µM sonidegib in the presence of LPS (1 µg/mL). At 0 and 24 h, the wound was observed, and images were obtained using a phase-contrast microscope (Nikon Instruments Inc., Melville, NY, USA). The degree of cell migration was described as the percentage of vehicle-treated control cells [5].

### 4.6. Enzyme-Linked Immunosorbent Assay (ELISA)

BV2 cells (2.5 *×* 10^5^ cells/well) were seeded into 24-well plates and treated with LPS (1 µg/mL) and sonidegib at the indicated concentrations for 24 h. After treatment, the cell culture medium was collected and centrifuged at 1500 rpm at 4 °C for 10 min. The supernatants were then stored at –80 °C until use. The production of IL-1β, IL-6, and TNF-α was measured using the respective ELISA kits (KomaBiotech, Seoul, Korea) in accordance with the manufacturer’s instructions.

### 4.7. Measurement of NO

BV2 cells (2.5 *×* 10^5^ cells/well) were seeded into 24-well plates and treated for 24 h with LPS (1 µg/mL) and sonidegib at the indicated concentrations. After treatment, the cell culture medium was collected and centrifuged at 1500 rpm at 4 °C for 10 min. The supernatants were then stored at –80 °C until use. The levels of NO were measured as nitrite by using Griess reagent (Promega Corporation, Madison, WI, USA) in accordance with the manufacturers’ instructions.

### 4.8. Immunocytochemistry

BV2 cells were immunostained to examine the NF-κB nuclear translocation, as reported previously [5]. In brief, cells (2.5 *×* 10^4^ cells/well) were seeded on coverslips placed on wells of 24-well plates, incubated at 37 °C for 24 h, and treated with LPS (1 µg/mL) and sonidegib (10 µM) for another 24 h. After the treatment, the coverslips were fixed using 4% paraformaldehyde at room temperature for 15 min, incubated with 1% Triton X-100 for permeabilization for 5 min, and blocked with 5% goat serum for 30 min. The cells were washed with PBS and incubated with the anti-NF-κB p65 antibody in the blocking solution at 4 °C overnight. On the following day, the cells were incubated with Alexa Fluor 488-conjugated anti-mouse secondary antibody and counterstained with DAPI in the dark at room temperature for 1 h. The coverslips were mounted with fluorescent mounting medium and sealed with nail polish to prevent drying and movement. Fluorescence images were obtained using a confocal microscope (Nikon Instruments Inc., Melville, NY, USA).

### 4.9. Animals

Animal studies were conducted following the protocol approved by the Institute of Laboratory Animal Research Center at Dongguk University, Korea (Approval number: IACUC-2021-009-1; Date of Approval: 10 May 2021). Six-week-old male ICR mice (approximately 30 g body weight) were purchased from the Orient Bio (Gyeonggi, Korea) and acclimatized to the environment with controlled ambient temperature of 20–25 °C, 48–52% relative humidity, and 12 h/12 h light/dark cycle (lights on 9:00 a.m.). Diet and water were provided ad libitum.

### 4.10. Treatment of Animals

**Figure 9:**
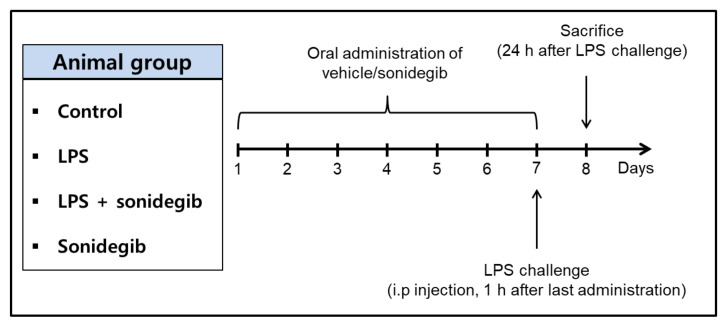
Animal experiment protocol. Mice were divided into four groups (n = 8 in each group): control, LPS, LPS + sonidegib, and sonidegib alone. Sonidegib (40 mg/kg, p.o.) or vehicle (ethanol/Tween 80 in water, p.o.) was administered once daily for 7 days. On the 7th day, LPS dissolved in sterile saline or vehicle (sterile saline) was intraperitoneally injected at a dosage of 3 mg/kg 1 h after the last administration of sonidegib. All mice were sacrificed at 24 h after LPS injection and brain tissues were obtained for the determination of IL-6 and TNF-α.

The mice were randomly divided into four groups (n = 8 in each group): control, LPS, LPS + sonidegib, and sonidegib alone. The mice in the LPS + sonidegib and sonidegib alone groups were orally administered with sonidegib dissolved in a solution containing 7.5% Tween 80, 17.5% ethanol, and 75% water at a dosage of 40 mg/kg body weight for 7 consecutive days. For the control and LPS groups, vehicle was administered instead. On the 7th day, LPS dissolved in sterile saline was intraperitoneally injected to the mice in the LPS and LPS + sonidegib groups at a dosage of 3 mg/kg 1 h after the last administration of sonidegib. The dose of LPS injection was determined based on our preliminary experiments. All animals were sacrificed after 24 h of LPS challenge (day 8), as illustrated in Figure 9. The brains were collected for determination of pro-inflammatory mediators. In brief, the mice were gently euthanized, and transcardial perfusion was performed with cold PBS. After decapitation, the brain was carefully extracted, homogenized in cold lysis buffer, and centrifuged at 14,000 rpm at 4 °C for 30 min. The collected supernatant was kept at −80 °C until use.

### 4.11. Determination of IL-6 and TNF-α in Brain Homogenates

The levels of IL-6 and TNF-α in the brain were measured using specific ELISA kits (KomaBiotech, Seoul, Korea). Briefly, serial dilutions of standards and samples were added to 96-well ELISA plates, followed by addition of biotinylated antibody. Then, the prepared solution of horseradish peroxidase-conjugated complex was added after rinsing with the wash buffer. The reaction was stopped by adding the stopping solution, and the absorbance of IL-6 and TNF-α was read at 450 nm.

### 4.12. Statistical Analysis

All experiments were performed at least three times and the results were expressed as the means *±* SEM. One-way analysis of variance (ANOVA) in Sigma Plot 12.5 software (Systat Software Inc., San Jose, CA, USA) was used to test the significant differences between groups. Differences were considered as significant with *p* < 0.05.

## 5. Conclusions

Among potential JNK3 inhibitors identified by structure-based virtual screening, sonidegib, an SMO antagonist approved for the treatment of basal cell carcinoma, was selected for further investigation. Our docking simulation and ^19^F NMR spectroscopy revealed that sonidegib could bind to the ATP binding site of JNK3 in an ATP-competitive manner with high affinity, showing a simulation score of −10.4 kcal/mol. In BV2 cells, sonidegib inhibited LPS-induced phosphorylation of JNK, confirming our virtual screening results. The LPS-induced phosphorylation of MKK4 and c-Jun was also inhibited by sonidegib. Moreover, the LPS-stimulated BV2 cell migration was markedly inhibited. It also suppressed the LPS-induced production of pro-inflammatory mediators, including IL-1β, IL-6, TNF-α, and NO, and the expression of pro-inflammatory enzymes, iNOS and COX-2, in BV2 cells. The anti-inflammatory effect of sonidegib was further verified by in vivo experiments. Furthermore, sonidegib inhibited the LPS-induced IκB phosphorylation, thereby blocking NF-κB nuclear translocation. These results indicated that sonidegib exerted anti-inflammatory and anti-migratory effects in LPS-treated BV2 cells and mice via the inhibition of JNK and NF-κB signaling. Based on our findings, sonidegib may have potential for drug repurposing to alleviate neuroinflammation associated with microglial activation in many brain disorders.

## Data Availability

Not applicable.

## Abbreviations

Aβ	amyloid beta
AD	Alzheimer’s disease
AMPPNP	adenylyl-imidodiphosphate
ATP	adenosine triphosphate
BBB	blood–brain barrier
CNS	central nervous system
COX-2	cyclooxygenase-2
FDA	U.S. Food and Drug Administration
IκB	inhibitory kappa B
IL	interleukin
iNOS	inducible nitric oxide synthase
LPS	lipopolysaccharide
MAPK	mitogen-activated protein kinase
MKK	mitogen-activated protein kinase kinase
MPTP	1-methyl-4-phenyl-1,2,4,6-tet-57 rahydropyridine
NF-κB	nuclear factor-kappa B
NMR	nuclear magnetic resonance
NO	nitric oxide
PD	Parkinson’s disease
PDB	Protein data bank
ROS	reactive oxygen species
SMO	smoothened
TNF-α	tumor necrosis factor-alpha
